# Outpatient Hysteroscopic Polypectomy—A Retrospective Study Comparing Rigid and Semirigid Office Hysteroscopes

**DOI:** 10.3390/diagnostics13050988

**Published:** 2023-03-05

**Authors:** Benito Chiofalo, Mauro Calandra, Valentina Bruno, Vincenzo Tarantino, Giovanni Esposito, Enrico Vizza, Giacomo Corrado, Giovanni Scambia, Ursula Catena

**Affiliations:** 1 Gynecologic Oncology Unit, Department of Experimental Clinical Oncology, IRCCS Regina Elena National Cancer Institute, 00128 Rome, Italy; 2 Division of Gynecologic Oncology, Department of Woman, Child and Public Health, Fondazione Policlinico Universitario A. Gemelli–IRCCS, Catholic University of Sacred Heart, 00168 Rome, Italy

**Keywords:** endometrial polyps, outpatient hysteroscopy, operative hysteroscopy, VAS score

## Abstract

Endometrial polyps are one of the most common pathological conditions in gynecology. Hysteroscopy is the gold standard for the diagnosis and treatment of endometrial polyps. The purpose of this multicenter, retrospective study was to compare patients’ pain perception during an operative hysteroscopic endometrial polypectomy in an outpatient setting with two different hysteroscopes (rigid and semirigid) and to identify some clinical and intraoperative characteristics that are related to worsening pain during the procedure. We included women that underwent, at the same time as an diagnostic hysteroscopy, the complete removal of an endometrial polyp (using the see-and-treat strategy) without any kind of analgesia. A total of 166 patients were enrolled, of which 102 patients underwent a polypectomy with a semirigid hysteroscope and 64 patients underwent the procedure with a rigid hysteroscope. No differences were found during the diagnostic step; on the contrary, after the operative procedure, a statistically significant greater degree of pain was reported when the semirigid hysteroscope was used. Cervical stenosis and menopausal status were risk factors for pain both in the diagnostic step and in the operative one. Our results confirm that operative hysteroscopic endometrial polypectomy in an outpatient setting is an effective, safe, and well-tolerated procedure and indicate that it might be better tolerated if a rigid rather than semirigid instrument is used.

## 1. Introduction

Hysteroscopy is the best method for the direct evaluation of the endometrial cavity of patients with a broad spectrum of gynecological pathologies, such as abnormal uterine bleeding, endometrial polyps, submucous myomas, infertility, mullerian abnormalities, endometrial hyperplasia, and cancer.

Endometrial polyps are among the most common pathological conditions in gynecology; their prevalence is estimated between 10% and 40% in symptomatic women and up to 12% in asymptomatic women [1]. Usually, the symptomatology related to endometrial polyps is abnormal uterine bleeding, vaginal bleeding during menopause, and infertility [2]. The risk of the cancerization of endometrial polyps and, consequently, the need for resection do not appear to be related to a polyp’s size; however, the risk of cancer seems to be higher in patients presenting uterine bleeding, aged > 60 years, with diabetes mellitus, with hypertension, and who use tamoxifen [3].

Nowadays, hysteroscopy represents the gold standard not only for diagnosis but even for the treatment of intra-uterine pathologies, which can be performed in outpatient or inpatient settings [4]. The advantages of the outpatient procedure are evident, given that it does not require cervical dilatation, an operating room, or hospitalization, thereby minimizing costs. On the contrary, the disadvantages are represented by the pain experienced during the procedure and by the lack of widespread surgical skills among the operators in this kind of surgery. The “see and treat” strategy, applied in the outpatient setting, perfectly integrates the operative step with the diagnostic procedure [5]. This has been enabled by the introduction of innovative miniaturized instruments (5 or 7 Fr), scopes with working channels and continuous flow systems, and intrauterine morcellator devices [6]. These instruments allow an operator to quickly and effectively perform many hysteroscopic procedures without increasing the patient’s discomfort [7].

Operative outpatient procedures, also called “office hysteroscopies”, are usually performed without any medication; in fact, there is no high-quality evidence that using local anesthetics, nonsteroidal anti-inflammatory drugs, or other medical approaches, either when compared amongst themselves or with a placebo, improves a patient’s compliance or reduces their pain scores during hysteroscopy [8].

It has been proven that the outpatient procedure is also less expensive in terms of direct and indirect costs when compared with an inpatient hysteroscopy, for which the latter is usually performed in an operative room under general or local anesthesia [9,10].

Polypectomy is the most common hysteroscopic operative procedure worldwide and many studies have proven its safety and efficacy both in general and in an outpatient setting [1,7]. The procedure can be performed with rigid and semirigid hysteroscopes, which are equipped with a working channel for the introduction of cold operative instruments and/or miniaturized radiofrequency devices. The choice of how to perform the procedure must account for different factors, such as the operator’s skills, patient compliance, the number and size of polyps, and the quality of the hysteroscopic imagery; furthermore, the correct choice of instrumentation is very important [11]. Due to the lack of similar studies in the literature, the aim of the present study was to compare two different methods of performing an outpatient hysteroscopic polypectomy with a rigid and a semirigid hysteroscope to test the safety and effectiveness of both procedures and establish which of the two treatments is superior.

## 2. Materials and Methods

This article reports the results of a multicenter, retrospective pilot study. The study was approved by the local Ethical Committee of the IRCCS “Regina Elena” National Cancer Institute of Rome (promotion center) and of the Fondazione Policlinico Universitario A. Gemelli—IRCCS of Rome (participant center) (RS n. 1778/22, DIPUSVSP-PD-01-231). The design, analysis, interpretation of data, drafting, and revision of the study followed the Helsinki Declaration’s Committee on Publication Ethics guidelines (http://publicationethics.org/ (accessed on 1 September 2022)), and the reporting of the study was conducted using observational, routinely collected health data statements [12], which are available through the Enhancing the Quality and Transparency of Health Research Network website (www.equator-network.org (accessed on 1 September 2022)). The study was unadvertised, and no remuneration was offered to participants. Each enrolled patient was informed about aims and procedures and signed an informed consent to facilitate data collection for research purposes.

The first goal of this study was to compare operative outcomes and patients’ pain perceptions with respect to outpatient operative hysteroscopic endometrial polypectomy among two different groups of patients treated with two different hysteroscopes (rigid and semirigid) to assess which technique is more effective and less painful; the second goal was to identify clinical and intraoperative characteristics related to increased pain during the procedure.

### 2.1. Patients: Inclusion and Exclusion Criteria

We retrospectively and consecutively included all women with hysteroscopic diagnosis of endometrial polyp that underwent, at the same time of the diagnostic hysteroscopy, an outpatient operative procedure for the complete removal of the polyp (using the see-and-treat strategy) with the two different hysteroscopes mentioned earlier from March 2021 to July 2022. The study took place at the IRCCS “Regina Elena” National Cancer Institute of Rome (promoting center) and at the CLASS Hysteroscopy Center of Fondazione Policlinico Universitario A. Gemelli—IRCCS of Rome (participant center). We excluded patients with multiple polyps and other concomitant intrauterine pathologies requiring an additional procedure, patients with final histological diagnosis of myoma and endometrial carcinoma, patients that postponed the procedure, patients treated with instruments other than those under study, and patients that underwent procedures under any type of analgesia and those performed by trainees or unexperienced surgeons.

### 2.2. Treatments & Instrumentation

Patients were divided into two groups: the first group underwent polypectomy with a semirigid office hysteroscope (Gynecare Versascope, GYN group), and the second group underwent the procedure using a rigid office hysteroscope (Storz 5 mm Bettocchi hysteroscope, BETT group). In the present study, the choice of the instrument depended on physician’s preferences.

Gynecare Versascope^TM^ is a 3.2 mm semirigid hysteroscope; it has a 1.9 mm optic (fiber optic) with a 0° viewing angle and a single-use outer sheath equipped with irrigation and suction channels and an additional expandable plastic channel for the insertion of 7 or 5 Fr operative instruments [13].

Bettocchi continuous-flow is an outpatient operating hysteroscope (Karl Storz SE & Co.Kg, Tuttlingen, Germany); it consists of a 2.9 mm optic (lens optic) with a 30° hole-oblique range of vision, an inner operating sheath for irrigation that is 4.3 mm in diameter, and an outer sheath for fluid aspiration that is 5 mm in diameter [13]. In both cases, the procedure was carried out without the use of any kind of analgesia or premedication, using a vaginoscopic approach, performing the so-called “no touch” technique, and using 5 Fr instruments (bipolar electrodes, scissors, and grasping forceps) for the operative procedure. For uterine distension, a sterile saline solution was used, with filling pressure of up to 100 mmHg, which was maintained using a manual or an automated pressure delivery system.

### 2.3. Patients’ Evaluation

We evaluated the following clinical parameters in both groups: age, menopause, and number of attempts, and type of delivery. Intra- and post-procedural parameters evaluated were stenosis of the cervical canal (based on the operator’s subjective impression, which was overcome solely by the rotation of the instrument), intra- and post-operative complications, and the size (largest diameter) and histologic type of the removed lesions. Individual pain sensation at two different times during the exam was recorded using a 10 cm visual analogue scale (VAS); each patient was asked to describe the intensity of pain during the hysteroscopy, after the diagnostic phase, and after the operative procedure.

### 2.4. Statistical Analysis

Kolmogorov–Smirnov test was used to analyze data distribution. Data were expressed as mean ± standard deviation (SD) and median ± range when appropriate. Mann–Whitney test or *t*-test were used for the analysis of continuous variables; additionally, Mann–Whitney test was used to compare VAS scores for all different groups between diagnostic and operative hysteroscopic steps since data were found to be non-parametric. *t*-test was used to confirm that the two groups were homogeneous in terms of clinical characteristics, since data were normally distributed, together with the Fisher’s exact test, which was used in the analysis of contingency tables for categorical data. Spearman correlation test was used to verify any correlation between polyps’ size and VAS 2. Significance was set at *p* ≤ 0.05. Statistical analyses were performed using GraphPad Prism ver. 9.0.0 (GraphPad Software, San Diego, CA, USA).

Post hoc power analysis was conducted to estimate the power of our sample size in order to detect meaningful differences between groups in the operative phase. The model by Noether et al. was applied [14].

## 3. Results

A total of 166 patients were enrolled from March 2021 to July 2022. Of these, 102 underwent a polypectomy with a semirigid hysteroscope (GYN group) in the promotion center, and 64 underwent the procedure with a rigid hysteroscope (BETT group) in the participant center. All the procedures were carried out by two surgeons experienced in hysteroscopy, namely, B.C. and U.C.

Both groups were homogenous in terms of clinical parameters, the number of patients with an intra-procedural finding of severe cervical stenosis, and the size and histologic type of the polyps; furthermore, all the removed lesions were pedunculated, not sessile (Table 1).

No intra- or post-operative complications were detected in the two groups. 

**Figure 1:**
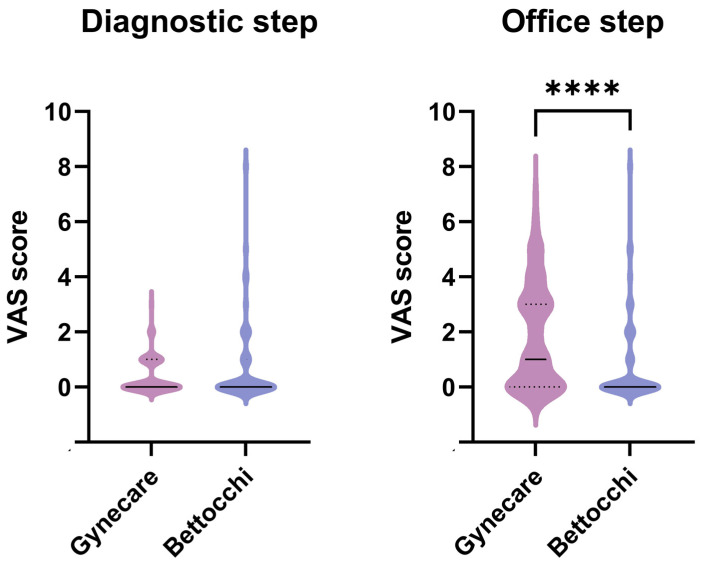
VAS score in the two groups during the diagnostic and the operative steps. **** Mann–Whitney test: *p* < 0.0001.

With regard to the VAS scores during the procedure, no differences were found after the diagnostic step (GYN median 0, range 0–3; BETT median 0, range 0–8); on the contrary, after the operative procedure, a statistically significant greater pain degree of was reported in the GYN group (GYN median 1, range 0–7; BETT median 0, range 0–8) (Figure 1).

After the data were processed to search for correlations between high VAS scores and clinical parameters or intraoperative findings, no correlation was found between VAS score and patients that underwent previous vaginal deliveries vs. those without previous vaginal delivery (*p* = 0.2 in the diagnostic step; *p* = 0.3 in the operative step) and previous cesarean section vs. those without previous cesarean section (*p* = 0.3 in the diagnostic step; *p* = 0.7 in the operative step). Conversely, cervical stenosis and menopause status were identified as statistically significant risk factors for pain both in the diagnostic step and in the operative one. Regarding the polyp size and histological type of the removed lesions, no correlations were found with VAS score during the operative step (r = 0,14; *p* = 0.07; *p* = 0.17) (Table 2).

Following post hoc power analysis, our sample resulted in a 37.6% level of power for detecting statistical differences between comparators, with an alpha error of 0.05.

## 4. Discussion

Hysteroscopy represents the gold standard for the diagnosis and treatment of intrauterine pathologies, and it should be considered for the treatment of endometrial polyps whenever possible [15,16,17].

Several types of hysteroscopes with different technological capabilities are currently available, and it is widely known that the smaller the diameter of the endoscope, the lower the degree of pain perceived; however, other factors must play a role in the genesis of pain during hysteroscopy.

The use of mini-hysteroscopes (3.3 mm with diagnostic sheath) seems to reduce the level of pelvic pain during this procedure [18].

Our study aimed to investigate pain perception during an office-based polypectomy among two groups of patients treated with a rigid and a semirigid hysteroscope; the results have shown that in the diagnostic phase, the procedure is painless and well-tolerated when using both rigid and semirigid hysteroscopes, each with two different diameters (3.6 vs. 5 mm). With regard to the operative procedure, the data show that the women who had a polypectomy with the rigid hysteroscope (BETT group) experienced less pain when compared with the GYN group.

The results regarding the GYN group, although higher than the BETT group, are still acceptable, and are similar to other data obtained by a previous study, wherein a Gynecare Versascope was used to perform outpatient hysteroscopies, and the mean intraoperative VAS score during the procedure was 1.2 [19].

The reason for such a difference could be related to the semirigid nature of the working channel of the Gynecare hysteroscope; indeed, the introduction of the operative instrument inside the expandable plastic channel provokes a sudden increase in the diameter of the hysteroscope; moreover, the position of the channel is uncontrollable due to its flexibility.

With the rigid hysteroscope, thanks to its 30° degree optical range, lateral movements of the instruments during the procedure can be avoided. This theory, although it cannot be adequately proven, can explain the increase in pain in the GYN group during the operative step.

Our secondary aim was to identify clinical characteristics and intraoperative parameters related to increased pain during the procedure. Our findings suggest that the correlation of pain with menopause and cervical stenosis was statistically significant; indeed, it is reasonable to think that cervical stenosis is more frequent in menopausal patients, for whom even partial synechiae render the transit of the instrument less comfortable and, therefore, more painful. These findings were confirmed both in the diagnostic and operative step, independently of the type of instrumentation used.

Furthermore, no correlations were found between the pain score and diameter of the lesion or the histologic type of the removed polyps. Our personal experience suggests that the surgical complexity is independent of the nature of the polyps, and it is likely that several factors play a role in this regard. The data regarding pain scores and polyp sizes are conflicting in the literature; however, few studies have considered the correlation with the histologic type [20,21].

There are conflicting data regarding the correlations between menopause, speculum placement, and a lack of previous vaginal delivery; according to a previous study, they seem to be associated with pain occurrence and intensity [22].

On the contrary, a Hungarian study found did not find any differences in the level of pain felt by the patients with different parity and menopausal statuses [23].

The majority of patients enrolled in the present study tolerated the outpatient procedure with low pain levels; this choice made it possible for them to receive a safe, efficient, and effective assessment and treatment, which avoided the disadvantages of a general anesthesia and hospital staying.

In a randomized trial, a significant patient preference for office-based hysteroscopy was reported, offering faster recovery times compared to an inpatient operative hysteroscopy and, of no small degree of importance, minimized procedural costs [24].

Conversely, other authors have observed that an office hysteroscopic polypectomy may be associated with a higher risk of failed or incomplete polyp removal [25,26].

In our centers, more than 90% of hysteroscopic polypectomies are performed in an outpatient setting, and, in the present study, all the procedures were successfully completed, with no residual polyp fragments.

The anxiety of patients undergoing hysteroscopy is mainly caused by the fear of feeling pain during the procedure; it is also associated with increased and persistent postoperative pain, and it seems to reduce patient satisfaction [27,28].

Obviously, it is very important to provide a patient with comfort and a friendly atmosphere while trying to medicalize the procedure as little as possible by using the vaginoscopic technique [13]. ‘No-touch’ vaginoscopic hysteroscopy is significantly faster to perform than the traditional technique and it is less painful, thereby reducing the likelihood of inducing a vasovagal reaction [22,29].

Communication and patient education have been proven to be effective tools with which to reduce preoperative anxiety; preoperative anxiety is reduced by the ability of doctors to answer patients’ questions, which also increases patients’ satisfaction [30].

In an outpatient setting, accurate counselling should be considered an essential part of the procedure, with the objective of achieving pain and anxiety reduction [31].

In this study, the patients were welcomed in both participating centers in dedicated clinics, with the presence of nurses adequately trained to support each woman. During the procedure, the surgeons provided support to the patient, involving her in the procedure and explaining the different steps of the procedure and any abnormalities, if present. These findings may have contributed to the very low pain levels reported during the hysteroscopic procedure.

Unsurprisingly, in the sample used in this study, even in cases where the VAS score was high, no vaso–vagal reactions occurred, and the use of atropine was not necessary in any case.

In a randomized perspective trial that evaluated the acceptance and feasibility of rigid hysteroscopes versus the flexible kind, the level of discomfort caused by the introduction of a rigid instrument was higher than that of the flexible one, even though rigid hysteroscopes provide superior optical qualities [19]. Although prospective and randomized, this study was limited to diagnostic hysteroscopy; it did not evaluate the execution of an operative procedure, which is the more painful phase of hysteroscopy, according to our data.

To the best of our knowledge, this is the first study that compares two different hysteroscopes for operative hysteroscopic polypectomy in an outpatient setting. In the literature, there are no studies that are similar to the ours; therefore, it is difficult, in a field that has been invaded by new, different optics and instruments over the last 10 years, each with different technologies, to find studies that can constitute a valid form of comparison.

Our data are not robust enough to draw any definite conclusions; however, despite the limitations of a retrospective series with low statistical power, our study showed that an operative hysteroscopic endometrial polypectomy in an outpatient setting with different types of instruments seems to be an effective, safe, and well-tolerated procedure. Moreover, it may be better tolerated if a rigid rather than a semirigid instrument is used. To clarify this last point, further studies and prospective randomized trials are required to establish the best way to perform endometrial polypectomy, while also considering surgeons’ and patients’ preferences.

Due to the advent of new technologies such as uterine morcellators, well-designed prospective studies are expected to be able to determine the best surgical techniques with which to perform outpatient endometrial polypectomy [32].

## Figures and Tables

**Table 1:** Clinical, intra-, and post-operative characteristics of enrolled patients.

	BETT	GYN	*p* Value
Total number	64	102	N.A.
Age (mean ± S.D.)	52.5 ± 14.2	51.5 ± 12.4	ns (* *p* = 0.6)
Parity (n pregnancies) (mean ± S.D.)	1.4 ± 1.3	1.5 ± 1.5	ns (* *p* = 0.7)
Cervical stenosis (%)	9.4	19.6	ns (^§^ *p* = 0.08)
Vaginal deliveries (%)	48.4	54.9	ns (^§^ *p* = 0.4)
Cesarean sections (%)	18.7	10.8	ns (^§^ *p* = 0.2)
Menopausal status (%)	48.4	50	ns (^§^ *p* = 0.9)
Polyp’s largest diameter (median (range))	15 (5–40)	10 (5–30)	ns (^ *p* = 0.06)
Polyp’s histologic type (FGP vs. HP)	74.6% vs. 25.4%	83.3% vs. 16.7%	ns (^§^ *p* = 0.17)

* *t*-test was used for comparisons between groups in terms of age and parity since data were normally distributed. ^ Mann–Whitney test was used for comparisons between groups in terms of polyp size. ^§^ Fisher’s exact test was used for comparisons between groups to assess differences in the categorical variables between the two groups. Significance was set at *p* ≤ 0.05. N.A—not applicable; BETT—rigid hysteroscope group; GYN—semirigid hysteroscope group; FGP—fibroglandular polyp; HP—hyperplastic polyp; ns—not significant.

**Table 2:** Correlation of VAS score with patients’ clinical and intra-operative characteristics.

	VAS in the Diagnostic Step		*p* Value	VAS In the Operative Step		*p* Value
	No Cervical Stenosis (n = 140)	Cervical Stenosis (n = 26)		No Cervical Stenosis (n = 140)	Cervical Stenosis (n = 26)	
Median (range)	0 (0–5)	1 (0–8)	*p* < 0.0001	0 (0–6)	3 (0–8)	*p* < 0.0001
	Fertile status (n = 84)	Menopause (n = 82)		Fertile status (n = 84)	Menopause (n = 82)	
Median (range)	0 (0–5)	0 (0–8)	*p* < 0.05	0 (0–5)	1 (0–8)	*p* < 0.05
	-	-		FGP (n = 133)	HP (n = 33)	
Median (range)	-	-		1 (0–8)	0 (0–7)	*p* < 0.65

VAS—visual analogue scale; FGP—fibroglandular polyp; HP—hyperplastic polyp.

## Data Availability

The data that support the findings of this study are available from the corresponding author, upon reasonable request.
